# QFT-Plus: a plus in variability? – Evaluation of new generation IGRA in serial testing of students with a migration background in Germany

**DOI:** 10.1186/s12995-016-0148-z

**Published:** 2017-01-05

**Authors:** J. Knierer, E. N. Gallegos Morales, A. Schablon, A. Nienhaus, J. F. Kersten

**Affiliations:** 1 University Medical Center Hamburg-Eppendorf (UKE), Center of Excellence for Health Services Research in Nursing (CVcare), Hamburg, Germany; 2 Institute for Statutory Accident Insurance and Prevention in the Health and Welfare Services (BGW), Principles of Prevention and Rehabilitation Department (GPR), Hamburg, Germany

**Keywords:** Latent tuberculosis infection, QFT-Plus, Variability, Serial testing, Students

## Abstract

**Background:**

Currently available Interferon-gamma release assays (IGRAs) show a considerable variability in serial testing for latent tuberculosis infection (LTBI).

This study offers first results for the new generation IGRA QuantiFERON-TB Gold Plus (QFT-Plus) introduced in 2015 in comparison with its predecessor QuantiFERON-TB Gold In-Tube (QFT-GIT) from serial testing of students with a migration background at German universities.

**Methods:**

Forty-one students were selected from a previous study. All students with a positive IGRA were asked and 11 agreed to participate in this cohort study. Additionally 30 students with negative IGRA results were selected by chance. Weekly testing with QFT-Plus and QFT-GIT was performed in all individuals over a 4-week period. IGRA variability was evaluated by calculating conversion and reversion rates.

**Results:**

From 41 participants a total number of 163 serial measurements were analyzed for each IGRA, leading to 122 possible IGRA changes each. QFT-Plus had four conversions and two reversions leading to a conversion rate of 4.3% (4 of 93 possible conversions, 95% CI 1.4–11.3%) and reversion rate of 6.9% (2 of 29 possible reversions, 95% CI 1.2–24.2%). QFT-GIT had 2 conversions and 1 reversion causing slightly lower rates with 2.2% conversions (2 of 91, 95% CI 0.4–8.5%) and 3.2% reversions (1 of 31, 95% CI 0.2–18.5%). Inconsistent IGRA results occurred in 4 subjects for QFT-Plus (8 stable positives, 29 stable negatives) and in 2 subjects for QFT-GIT (9 stable positives, 30 stable negatives). Agreement between the two IGRAs was 95.1% (*κ* = 0.89). Variance attributed to the individuals was low (QFT-Plus: ICC = 0.88).

**Conclusion:**

This study confirms occurrence of conversions and reversions for the new QFT-Plus in serial testing of a high-risk cohort in a low-incidence setting with improbable new TB contact during the study. QFT-Plus conversion and reversion rates were slightly higher than for the QFT-GIT but overall they were lower for both IGRAs than in other studies that investigated IGRA variability.

**Electronic supplementary material:**

The online version of this article (doi:10.1186/s12995-016-0148-z) contains supplementary material, which is available to authorized users.

## Background

Since its first approval by the U.S. Food and Drug Administration (FDA) in 2001 [1] hopes were high for the Interferon-gamma release assay (IGRA) as a new method to test for latent tuberculosis infection (LTBI).

Meanwhile, for over a decade IGRAs have played an important role in global tuberculosis control as they offer a convenient way to screen for LTBI. They are increasingly used in high-income countries instead of the tuberculin skin test (TST) for LTBI-testing.

IGRAs have major advantages over the TST, such as delivering results after 24 h with no need for a second appointment, not being affected by the examiner’s subjectivity that may influence TST interpretation, not struggling as an in vitro test with the problem of boosting in subsequent tests, using a positive control to facilitate interpretation in immunocompromised subjects and not cross-reacting with most nontuberculous mycobacteria (NTM) or BCG vaccine strains [2]. The latter improvement should lead to a higher specificity in BCG vaccinated subjects while maintaining a good sensitivity like that of the TST [3–5].

Beside their advantages over TST, IGRAs tend to show a high variability with high rates of conversions and reversions in serial testing, which was shown to be present in low as well as high TB incidence settings [6, 7]. Tagmouti et al. notice in a systematic review that IGRA test variability is higher than TST variability [8]. In settings where serial IGRA testing for LTBI is done, the elevated test variability leads to uncertainty whether chest X-ray or preventive chemotherapy is needed in the event of a positive test result with the knowledge that reversions tend to occur more frequently than conversions [8–11].

Following results from recent studies, several guidelines step back from promoting IGRAs as the standalone test for serial LTBI screening. Canada’s 2013 guideline update recommends TST rather than IGRA for testing low-risk individuals, noting that IGRA is not recommended for serial testing for populations with ongoing exposure [12]. The 2010 updated U.S. guidelines allow either TST or IGRA as the method of choice for serial testing, pointing out that using IGRA might lead to higher conversion rates making it difficult to identify IGRA changes that truly represent a new infection [13]. In Europe the ECDC guidelines allow IGRA as a baseline test, likewise pointing out problems of increased test variability offering a two-step approach in order to increase specificity [14].

Pai et al. postulate the simple dichotomous definition of IGRA test results to be inappropriate as it causes higher conversion rates than what the annual TB infection risk in low-incidence settings would lead to expect [15]. Several studies suggest the use of a borderline zone in order to avoid unnecessary X-rays and preventive chemotherapy [9, 16–20].

Until recently two IGRAs were commercially available, QuantiFERON-TB Gold In-Tube (QFT-GIT) (Qiagen, Hilden, Germany) and T-SPOT.TB (Oxford Immunotec, Abingdon, UK) that have been subjects of interest for many studies. In 2015 Qiagen updated its IGRA lineup, now offering a new generation of QFT-GIT: QuantiFERON-TB Gold Plus (QFT-Plus). According to the manufacturer the QFT-Plus offers higher sensitivity and specificity in patients at highest risk for TB-infection and in immunocompromised patients [21]. The aim of this study was to see if this new generation IGRA offers a plus in areas where present IGRA products tend to struggle. We are aware of only one paper, published independently from the producer of QFT-Plus which investigates sensitivity and specificity of the new generation IGRA [22]. In particular, so far, no data is available on test variability of new generation QFT-Plus in serial testing. Therefore, the primary objective of our study was to evaluate QFT-Plus test variability in a direct head-to-head comparison of QFT-GIT and QFT-Plus in a cohort of students and young professionals with a migration background currently studying at a German university or technical college.

## Methods

### Study design and subjects

We recruited a total of 41 participants from our partner study in which students and young professionals with migration background were screened for LTBI using both IGRAs (QFT-GIT and QFT-Plus) (Gallegos Morales EN, Knierer J, Schablon A, Nienhaus A, Kersten JF: Prevalence of Latent Tuberculosis Infection among foreign students tested with QuantiFERON-TB Gold In-Tube and QuantiFERON-TB Gold Plus, submitted). All students that were screened positive in at least one of the two IGRAs at baseline were asked to participate in this cohort study. 11 out of 13 students with positive baseline results agreed to participate. Another 30 participants with negative baseline results in both IGRAs were selected by chance. The study was conducted at universities and technical colleges in Lübeck, Germany, between February 2016 and March 2016.

Inclusion criteria were an age of at least 18 years, student status or having completed studies at a German university, and a migration background. For fulfilling the migration background criteria, participants must have been foreign born in a country with a WHO-estimated tuberculosis incidence of at least 10 incident cases per 100,000 inhabitants per annum (hereinafter: ≧10:100,000). If born in a country with lower incidence, having a nationality of a country with a TB incidence of at least 10:100,000 allowed participation [23]. People without a migration background fulfilling the nationality criteria but only having that nationality because of their parents’ nationality, were excluded.

Some IGRA studies tend to exclude people that have had a TST during the previous 3–6 months [17, 24]. In our study a recent TST application was not an exclusion criterion because many students, especially medicine students or students of natural sciences, do a TST during classes at the university. So the timeframe of the last TST application was queried in the questionnaire. None of the participants had a TST application during the ongoing study.

Exclusion criteria were withdrawal of consent, ongoing active TB infection or treatment for LTBI, insufficient Interferon-gamma (IFN-γ) production in the positive control at the first screening visit, and absence from more than one study visit.

### Questionnaire items

The standardized questionnaire was offered in two languages: German and English. It covered information on the participants’ study subject, work area, nationality and country of birth, time living in Germany permanently, immunosuppression caused by disease or current medication, information on contact with people with active tuberculosis, and specification of that contact. In detail we asked for: recent participation in tuberculosis screening and recent chest X-ray results; previous TST or IGRA results as well as BCG vaccination status (anamnestic and/or verification by vaccination scars); own TB history and history of treatment for active TB or LTBI.

### Diagnostic methods and data collection

The new generation IGRA QFT-Plus carries a lot of its predecessors’ characteristics but uses two antigen tubes (TB1 and TB2) instead of one in the QFT-GIT. As in the QFT-GIT, T-lymphocyte secreted IFN-γ is quantitatively measured by ELISA technique after in vitro stimulation with antigens highly specific for Mycobacterium tuberculosis.

Both tubes contain the antigens ESAT-6 and CFP-10 (TB 7.7 additionally used in the QFT-GIT tube is no longer present in QFT-Plus tubes). The TB1 tube is designed to elicit cell-mediated immune (CMI) responses from CD4^+^ T-helper lymphocytes whereas the TB2 tube additionally contains a set of peptides targeted to the induction of CMI responses from CD8^+^ cytotoxic T-lymphocytes [25]. The latter might potentially help to distinguish active from latent TB and should improve sensitivity in immunocompromised patients e.g. children or HIV co-infected patients [21, 25].

Collection of all blood samples and care for participants were performed by two study physicians (JK, ENGM). Weekly testing (7 +/- 1 days) with QFT-Plus and QFT-GIT was performed in all individuals over a 4-week period. Participants were given individual appointments to make sure that the blood was taken at the same time of day on each visit.

As some studies suggest that variation of blood volume in the QFT tubes might be a reason for increased test variability [26], the blood was first collected in tubes with lithium heparin, expecting that a subsequent in-lab transfer of the blood in the QFT tubes would secure transfer of the accurate blood volume. The blood samples were transported to the laboratory within 8 h after blood collection. QFT tubes were then inoculated manually by professional medical technicians and directly incubated in strict adherence to the manufacturer’s instructions within one hour after arrival of the samples at the laboratory.

IFN-γ ELISAs were performed directly after the incubation on all blood samples.

As suggested in the manufacturer’s instructions QFT-GIT results were considered positive if the IFN-γ value was ≧ 0.35 IU/ml after correction for the negative control. QFT-Plus results were considered positive if the IFN-γ response after correction for the negative control was ≧ 0.35 IU/ml in one of the two TB antigen tubes (TB1 or TB2) or if both tubes (TB1 and TB2) showed ≧ 0.35 IU/ml.

Measurement resolution and laboratory restrictions led to a truncated interval of IFN-γ values: IFN-γ values > 10 IU/ml were leveled at 10 IU/ml; IFN-γ values <0.01 IU/ml or negative values were leveled at 0.00 IU/ml.

Conversion was defined as the change from a negative result (<0.35 IU/ml) in the previous visit to a positive result (≧0.35 IU/ml) in the next visit. In line with the above definition for QFT-Plus interpretation, it was sufficient if the change from negative results (<0.35 IU/ml) in both TB antigen tubes to a positive result (≧0.35 IU/ml) was achieved in one of the two TB antigen tubes to count as a conversion. Reversion was defined as the change from a positive result to a negative result.

All participants were offered advice and explanation of their personal test results by the study physicians.

To rule out the possibility of active TB, participants with a positive test result in more than one study visit were advised to take a chest X-ray if not recently performed in context of TB screening. Participants with prior contact to a person with active TB during the last 2 years and positive results qualified for preventive chemotherapy. They were sent to pulmonary consultants for a second opinion and for planning and supervision of the therapy if suitable.

### Statistical analysis

Categorical data is presented as counts and corresponding rates, continuous variables are presented as means, standard deviations (SD) and ranges where appropriate.

Agreement between the two test methods (QFT-GIT and QFT-Plus) was analyzed using Cohen’s kappa coefficient. The variance attributed to the individuals, i.e. test reliability in the individual, was investigated for the QFT-Plus with the intraclass correlation coefficient (ICC) in a 1-way design. An ICC of >0.8 is regarded as indicative for a high agreement and therefore a low intraindividual variance [27].

Data evaluation was performed using SPSS Version 22 (IBM Corp., Armonk, NY USA).

## Results

### Study population and risk factors for IGRA-positivity at baseline

The characteristics of our study population and baseline QFT-Plus results are presented in Table 1. Participants had a mean age of 25.5 ± 3.6 (range: 20 to 36) years, average time living in Germany was 3.8 ± 3.8 years (range: 4 months to 23.6 years), and 22 participants were born in a country with a TB incidence of ≧ 125:100,000. Participants’ country of birth or country that allowed participation included all six WHO-regions: Africa (including: Cameroon, Ghana, Kenya, Nigeria, South Africa and United Republic of Tanzania), The Americas (including: Ecuador, Guatemala, Honduras and Mexico), South-East Asia (including: India, Indonesia and Nepal), Europe (including: Belarus, Lithunia, Russian Federation, Tajikistan and Ukraine), Eastern Mediterranean (including: Syrian Arab Republic and Yemen) and Western Pacific (including: China, Mongolia, Philippines, Republic of Korea and Vietnam).

**Table 1:** Description of study population and frequencies of QFT-Plus results at baseline

Variable		QFT-Plus positive^a^	QFT-Plus negative^a^
	N (Col-%)	N (Row-%)	N (Row-%)
Gender			
Female	22 (53.7)	3 (13.6)	19 (86.4)
Male	19 (46.3)	6 (31.6)	13 (68.4)
Age			
20–25 years	26 (63.4)	3 (11.5)	23 (88.5)
26–36 years	15 (36.6)	6 (40.0)	9 (60.0)
Country of birth Classified by TB-incidence			
< 125:100 000	19 (46.3)	1 (5.3)	18 (94.7)
≥ 125:100 000	22 (53.7)	8 (36.4)	14 (63.6)
Classified by WHO-regions			
Africa	9 (22.0)	4 (44.4)	5 (55.6)
The Americas	6 (14.6)	0 (0.0)	6 (100.0)
South-East Asia	9 (22.0)	2 (22.2)	7 (77.8)
Europe	10 (24.4)	0 (0.0)	10 (100.0)
Eastern Mediterranean	2 (4.9)	0 (0.0)	2 (100.0)
Western Pacific	5 (12.2)	3 (60.0)	2 (40.0)
Time living in Germany			
< 2 years	15 (36.6)	3 (20.0)	12 (80.0)
2–10 years	25 (61.0)	6 (24.0)	19 (76.0)
> 10 years	1 (2.4)	0 (0.0)	1 (100.0)
Contact to TB			
Yes	19 (46.3)	6 (31.6)	13 (68.4)
No	22 (53.7)	3 (13.6)	19 (86.4)
Type of TB contact			
Relatives	8 (19.5)	5 (62.5)	3 (37.5)
Friends/acquaintances	3 (7.3)	0 (0.0)	3 (100.0)
Professional	6 (14.6)	0 (0.0)	6 (100.0)
Other type	2 (4.9)	1 (50.0)	1 (50.0)
TB in own history			
Yes	1 (2.4)	1 (100.0)	0 (0.0)
No	40 (97.6)	8 (20.0)	32 (80.0)
BCG-vaccination			
Yes	18 (43.9)	2 (11.1)	16 (88.9)
No	4 (9.8)	3 (75.0)	1 (25.0)
unknown	19 (46.3)	4 (21.1)	15 (78.9)
Previous TST			
Positive	3 (7.3)	2 (66.7)	1 (33.3)
Negative	5 (12.2)	0 (0.0)	5 (100.0)
Unknown	3 (7.3)	0 (0.0)	3 (100.0)
No TST	30 (73.2)	7 (23.3)	23 (76.7)

^a^QFT-Plus result at baseline

Nineteen participants reported contact to a person with active TB of which 6 (32%) had a positive QFT-Plus at baseline with five out of six (83%) defining the TB contact as a family member. 11 participants declared a TST application, the average time since last TST was 5.7 years; two of these reported a TST application during the six months before inclusion in the study (in both cases one month beforehand). Both had consistent IGRA results on all four visits with full concordance between the two IGRAs; one had consistently negative and the other showed consistently positive IGRA results. Being consistently positive this participant was classified as true positive. 30 participants were never tested with TST.

One IGRA-positive participant had a personal history of active TB. On inclusion in our study this student was smear- and culturally negative after completion of antituberculosis chemotherapy. None of the participants showed signs of ongoing active TB infection at the time of inclusion in the study.

### IGRA positivity throughout the study and test intervals

All 41 participants turned up for their weekly appointments, resulting in 164 blood samples.

On visit 3, the laboratory was unable to analyze one blood sample. This led to a total of 163 valid IFN-γ values for each of the two IGRAs, i.e. four serial measurements at weekly intervals for 40 participants and three measurements for one participant.

In one case (0.8%) the interval between tests was 6 days, 7-day interval in 107 cases (87.7%), 8-day interval in 13 cases (10.7%), and – because of the not analyzable blood sample – a 14-day interval in one case (0.8%).

All 11 recruited participants with initial IGRA positivity remained positive throughout the study in at least one of the two IGRAs. A 12th participant only showed one positive QFT-Plus result on visit 3 but reverted on the final visit. All 12 participants with IGRA positivity throughout the study had an LTBI risk due to their migration background: 11 participants with positive IGRA results were born in a country with a TB incidence of ≧125:100,000 and one positive participant was born in China which is classified as a high TB burden country by the WHO [28]. Over the whole study period nine participants showed stable positive QFT-GIT results and 30 had stable negative QFT-GIT results. For the QFT-Plus, eight participants were stable positive and 29 participants were stable negative. During the 4-week study period a total number of 42 positive results in the QFT-GIT and 40 positive results in the QFT-Plus were collected.

(Individual IGRA results and IFN-γ values for all participants throughout the study are shown in the table in the supplement [see Additional file 1]).

### Reversions and conversions

Reversions and conversions occurred in four participants with inconsistent results, i.e. inconsistency of dichotomous IGRA test decisions during the study period (Table 2). All four showed inconsistent QFT-Plus results and only two of them also showed inconsistent QFT-GIT results.

**Table 2:** Participants with inconsistent IGRA results in the weekly blood collections

Sex	No.	Age	IGRA	Visit 1	Visit 2	Visit 3	Visit 4	Overall trend
M	9	32	QFT-Plus	Neg. (0.15/0.19)	Neg. (0.17/0.14)	Neg. (0.14/0.09)	Pos. (0.34/0.40)	Inc.
			QFT-GIT	Pos. (0.40)	Pos. (0.62)	Pos. (0.66)	Pos. (0.65)	Pos.
M	10	20	QFT-Plus	Neg. (0.24/0.20)	Pos. (0.50/0.47)	Pos. (0.55/0.73)	Pos. (0.79/0.69)	Inc.
			QFT-GIT	Pos. (0.35)	Neg. (0.31)	Pos. (0.46)	Pos. (0.53)	Inc.
M	11	23	QFT-Plus	Pos. (0.32/0.64)	Neg. (0.11/0.12)	Pos. (0.46/0.40)	Pos. (0.56/0.61)	Inc.
			QFT-GIT	Neg. (0.18)	Pos. (0.52)	Pos. (0.47)	Pos. (0.63)	Inc.
M	12	25	QFT-Plus	Neg. (0.08/0.08)	Neg. (0.05/0.14)	Pos. (0.16/0.36)	Neg. (0.21/0.24)	Inc.
			QFT-GIT	Neg. (0.04)	Neg. (0.09)	Neg. (0.09)	Neg. (0.14)	Neg.

**Fig. 1:**
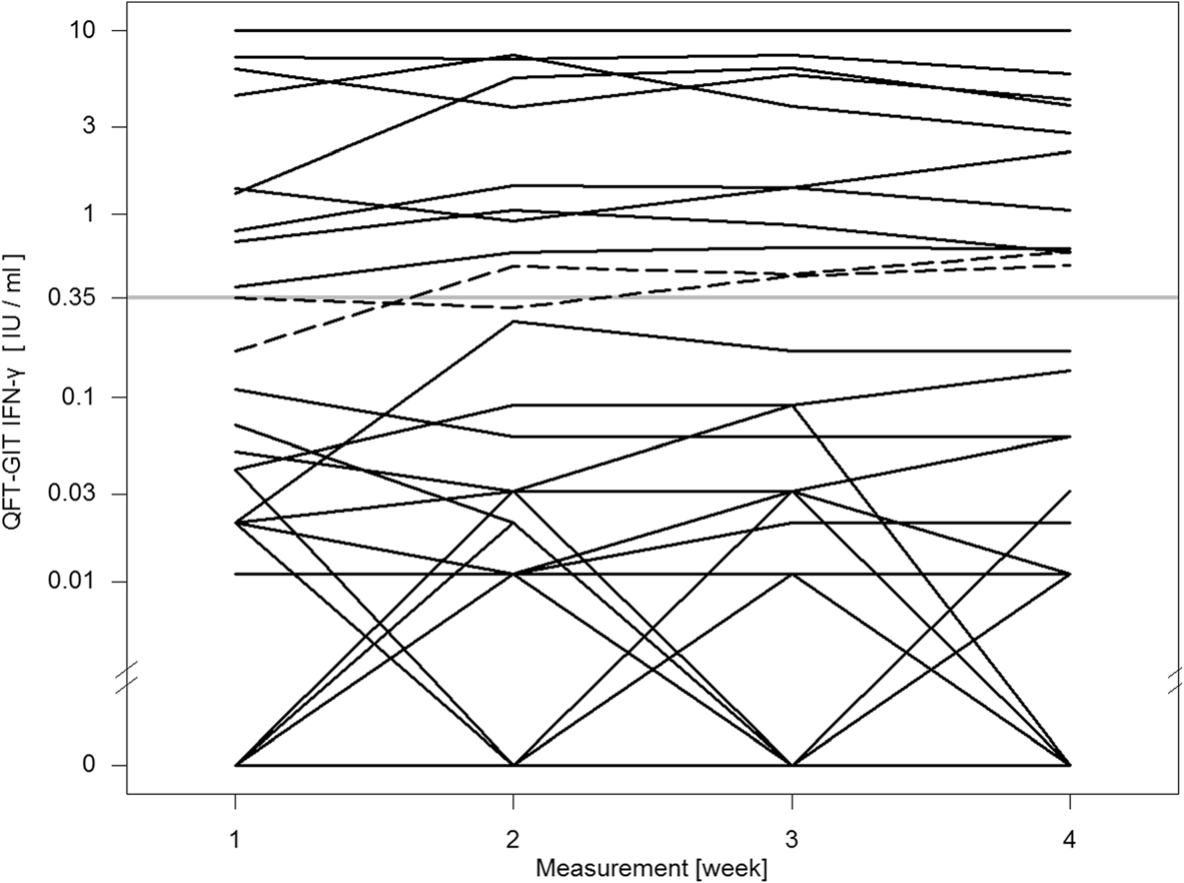
QFT-GIT continuous IFN-γ courses of the individual participants during the four study weeks

**Fig. 2:**
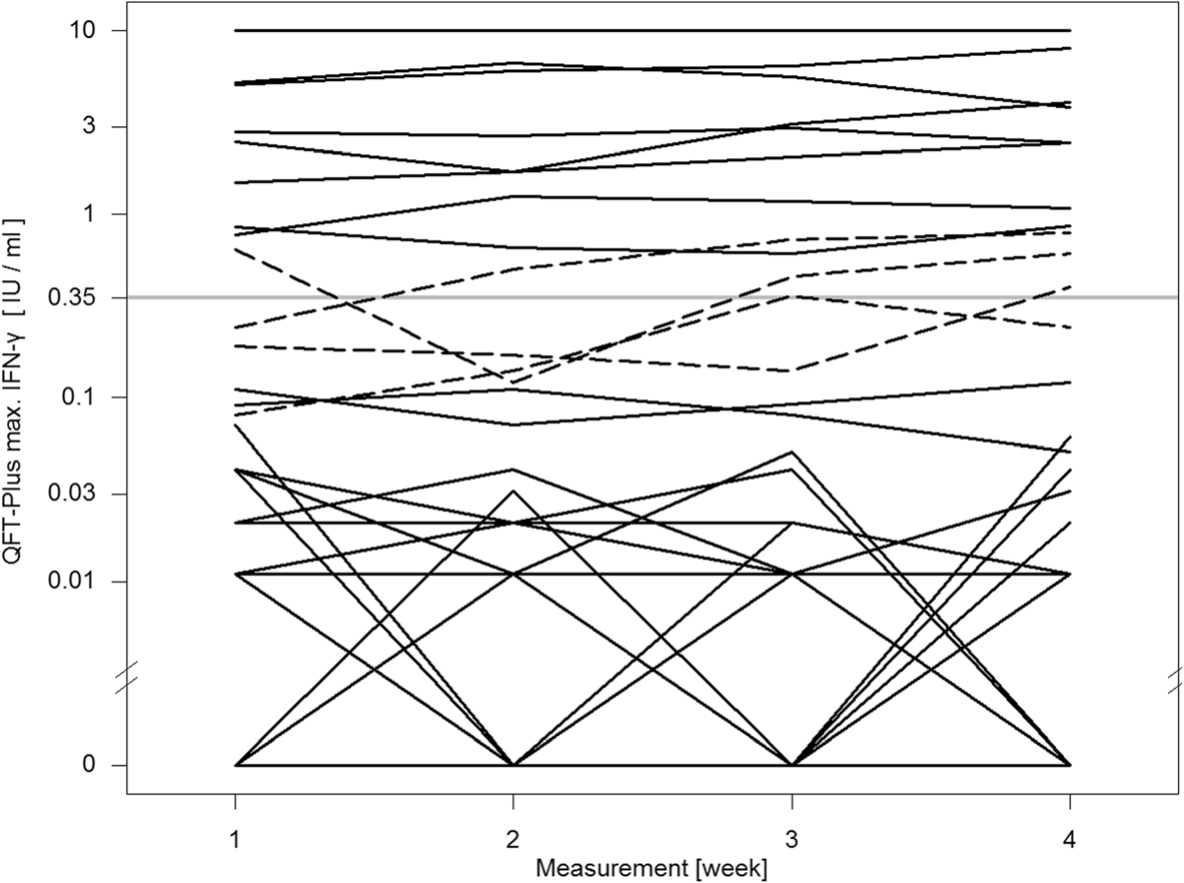
QFT-Plus continuous IFN-γ courses using maximum IFN-γ value of the two antigen tubes

**Fig. 3:**
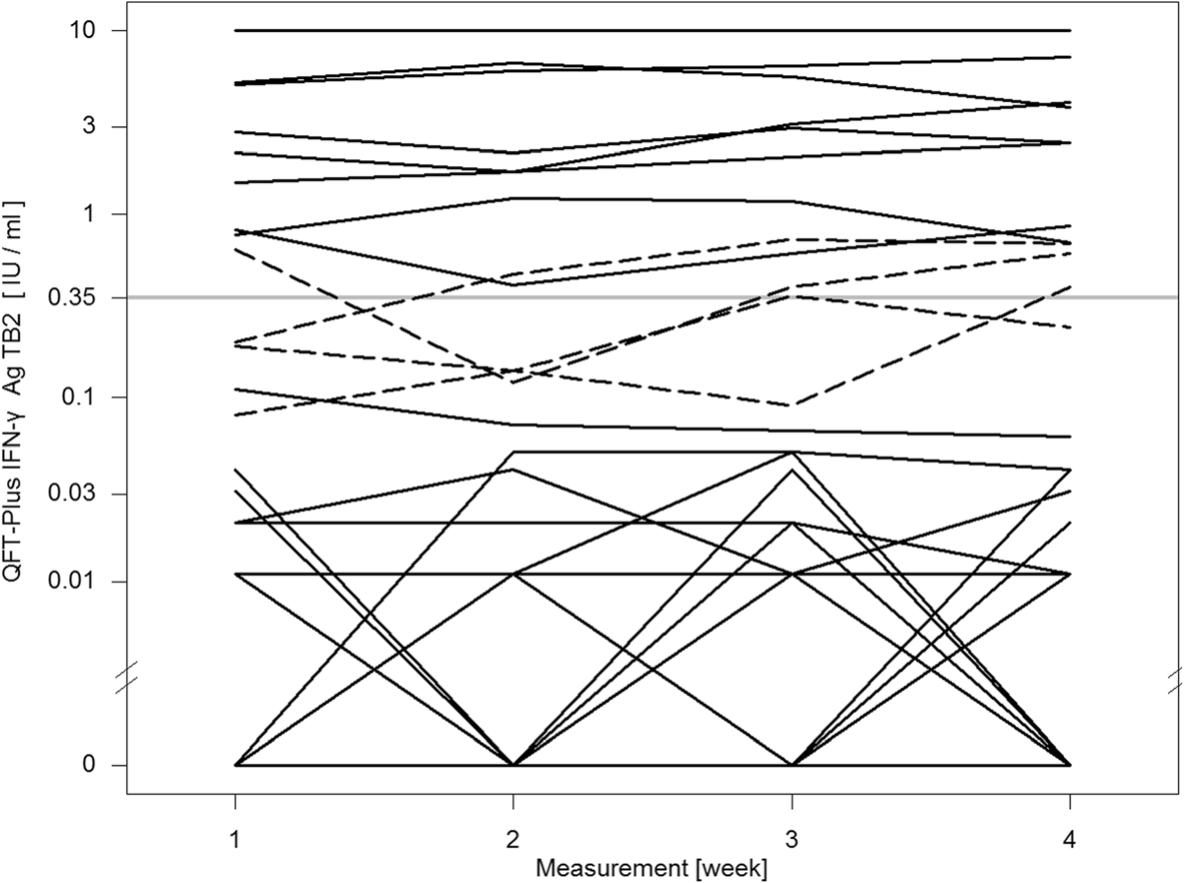
QFT-Plus continuous IFN-γ courses during the four study weeks of TB Antigen Tube 2 (TB2)

**Fig. 4:**
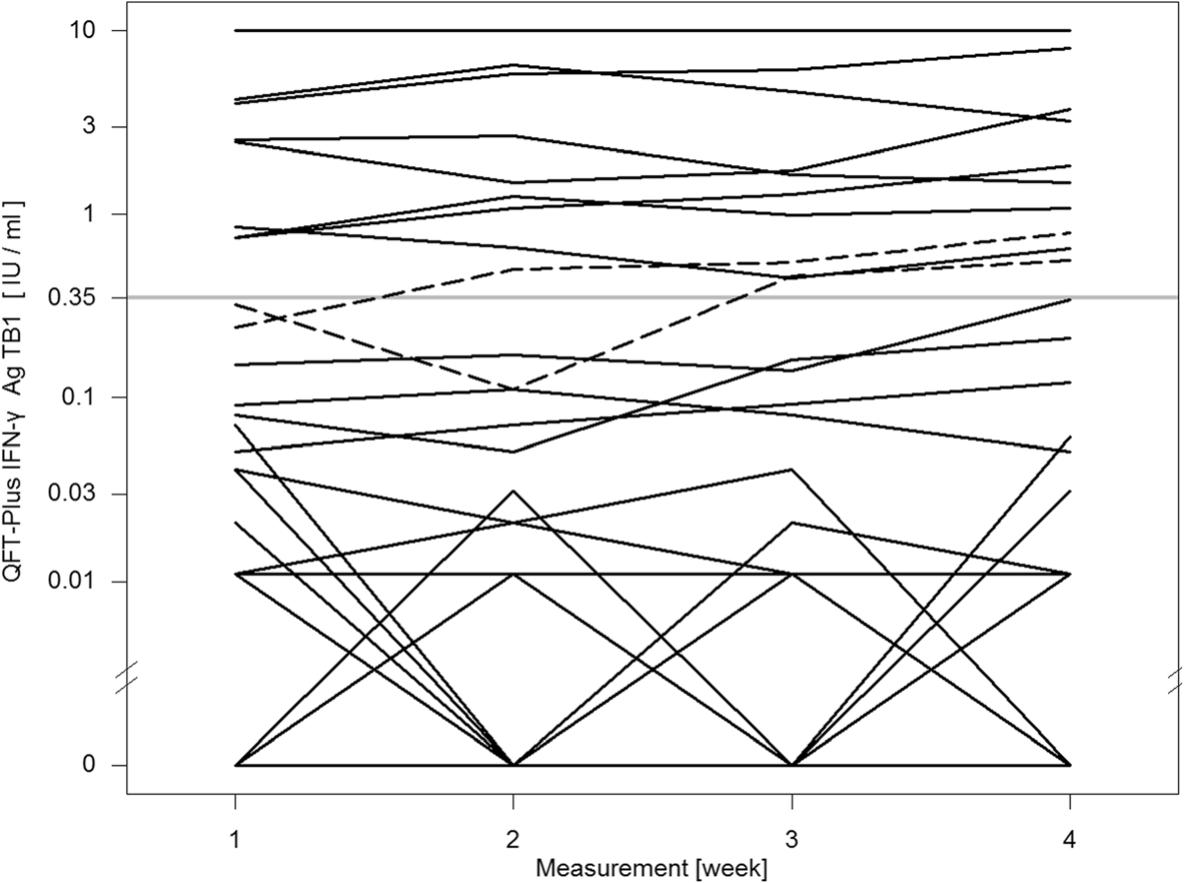
QFT-Plus continuous IFN-γ courses during the four study weeks of TB Antigen Tube 1 (TB1)

For the QFT-GIT two conversions and one reversion were detected. One conversion was stable, the other conversion and reversion were caused by a fluctuating result: a reversion followed by a reconversion on the subsequent visit (pos-neg-pos-pos) (Fig. 1). The QFT-Plus had four conversions and two reversions of which one conversion and one reversion were based on fluctuating results (pos-neg-pos-pos and neg-neg-pos-neg) (Fig. 2). In the QFT-Plus two of the conversions and two reversions occurred only because of the IFN-γ measurement crossing the cutoff in tube TB2 (Fig. 3) and not in tube TB1 (Fig. 4).

All inconsistent results of the QFT-GIT occurred in the same participants that showed inconsistent QFT-Plus results during the study period.

The conversions and reversions in both IGRAs took place within a range close to the cutoff. For the QFT-GIT the lowest IFN-γ value that showed a conversion was 0.18 IU/ml, for QFT-Plus tube TB1 it was 0.11 IU/ml and tube TB2 0.09 IU/ml. The highest IFN-γ value that showed a reversion was 0.35 IU/ml for QFT-GIT and 0.64 IU/ml for tube TB2 of QFT-Plus. No QFT-Plus reversion was caused by crossing of the cutoff in tube TB1.

### IGRA variability

**Fig. 5:**
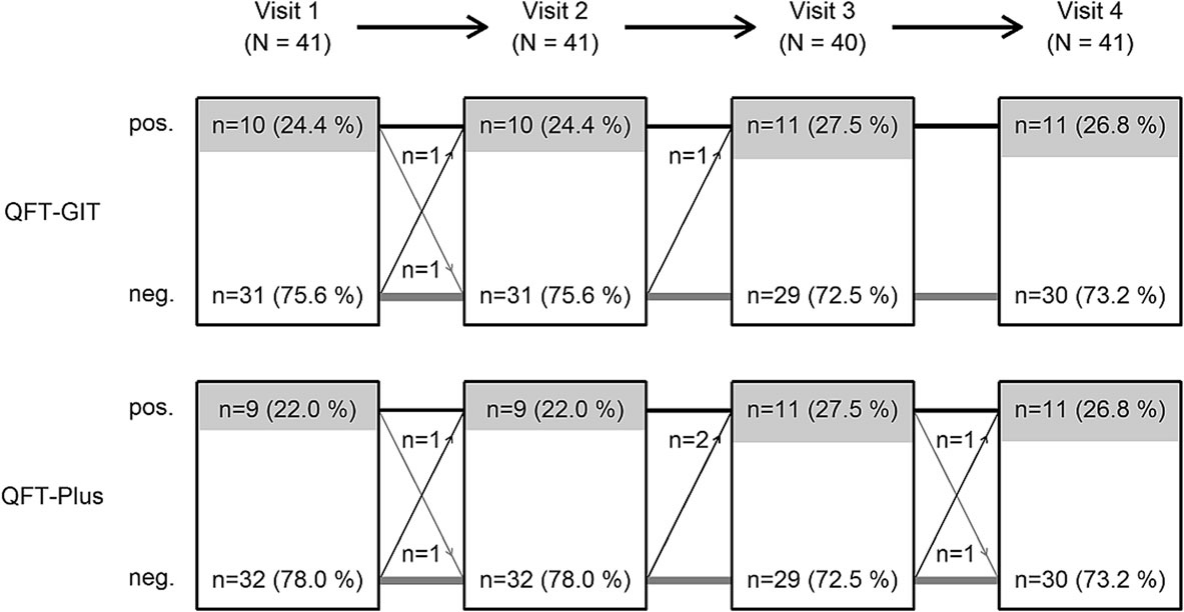
Flow chart of study population and IGRA results

For each participant four serial measurements at weekly intervals led to a possibility of reversions and conversions on three occasions. Overall, 163 valid IFN-γ values were collected for each IGRA. On the last visit 41 blood samples were analyzable, leaving 122 valid IFN-γ values for each IGRA that were followed by a subsequent result and therefore feasible for a change of the dichotomous IGRA test result. Total numbers for every visit with changes of IGRA results can be seen in the flow chart Fig. 5. With a total number of 42 positive QFT-GIT results of which 11 occurred on the final visit, 31 positive results were followed by a subsequent result and therefore feasible for reversion. Using the number of the above QFT-GIT reversions and conversions, a reversion rate of 3.2% (1 of 31 possible reversions, 95% CI 0.2–18.5%) and correspondingly calculated conversion rate of 2.2% (2 of 91 possible conversions, 95% CI 0.4–8.5%) was present in our study. The QFT-Plus had a reversion rate of 6.9% (2 of 29, 95% CI 1.2–24.2%) and a conversion rate of 4.3% (4 of 93, 95% CI 1.4–11.3%).

### Concordance between the two IGRAs and Intraclass Correlation (ICC)

On visits 1 and 2, in 92.7% of results (38 of 41 subjects) of the two IGRAs were concordant, 95% on visit 3 (38 of 40) and on the final visit the IGRA results were concordant in all of the participants (41 of 41), leading to an overall agreement between the two IGRAs of 95.1% (155 of 163); Kappa: 0.89 (Table 3).

**Table 3:** Concordance between the two IGRAs at each study visit

Visit No.	No. of valid results	Pos. QFT-Plus *n* (%)	Pos. QFT-GIT *n* (%)	Discordant results *n* (%)	Agreement between IGRAs
1	41	9 (22.0)	10 (24.4)	3 (7.3)	92.7%
2	41	9 (22.0)	10 (24.4)	3 (7.3)	92.7%
3	40	11 (27.5)	11 (27.5)	2 (5.0)	95.0%
4	41	11 (26.8)	11 (26.8)	0 (0.0)	100%
Total	163	40 (24.5)	42 (25.8)	8 (4.9)	95.1% (*κ* = 0.89)

**Fig. 6:**
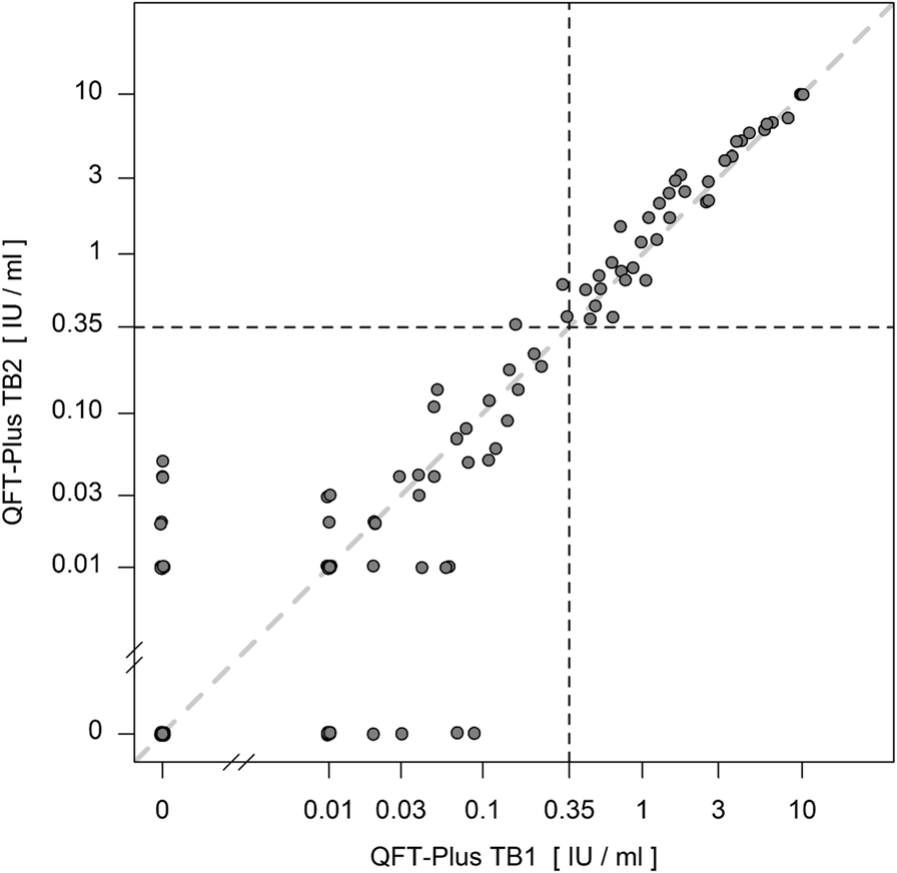
Concordance between the QFT-Plus tubes TB1 and TB2

**Fig. 7:**
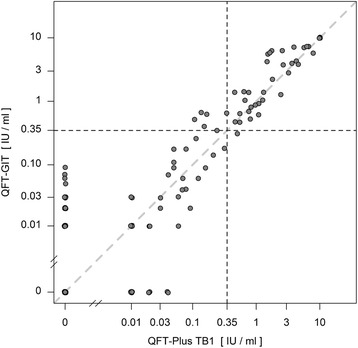
Concordance between QFT-GIT and QFT-Plus tube TB1

**Fig. 8:**
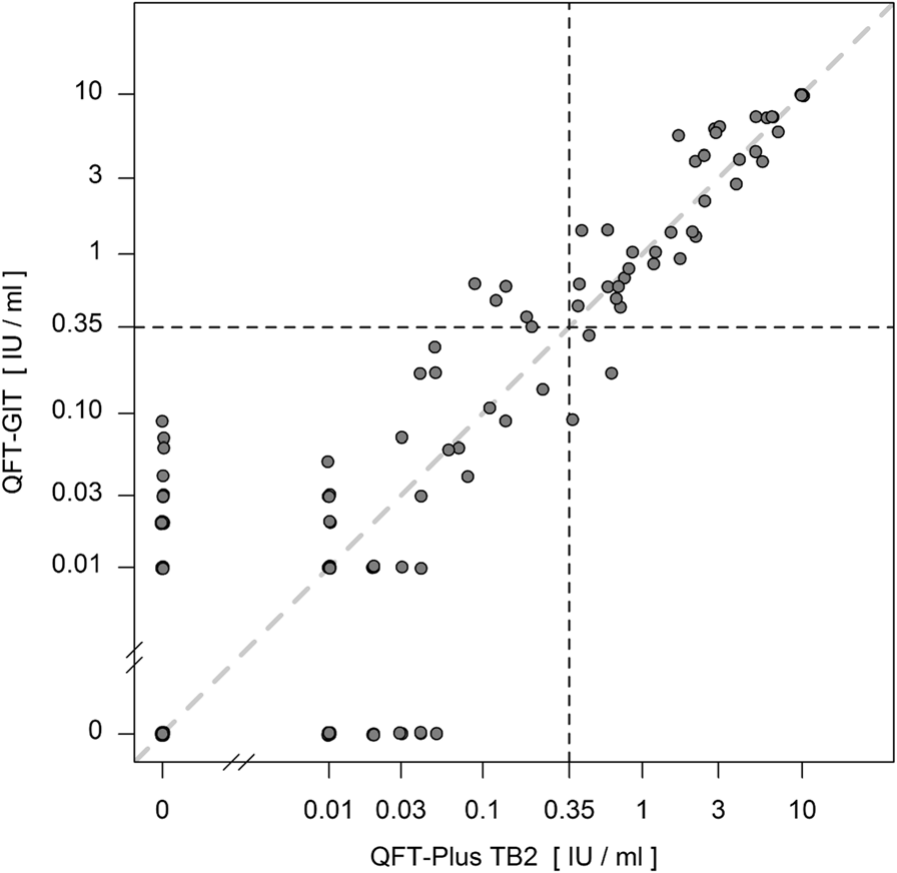
Concordance between QFT-GIT and QFT-Plus tube TB2

Figure 6 compares values of the two QFT-Plus tubes TB1 and TB2, showing that in three occasions QFT-Plus positivity was based on crossing of the manufacturers cutoff (0.35 IU/ml) only in tube TB2 whereas TB1 stayed under the cutoff. Figure 7 compares values of the QFT-GIT with QFT-Plus tube TB1 values. On six occasions the QFT-GIT showed values over the cutoff while the TB1 value was under the cutoff. Once the TB1 value was higher than the cutoff while QFT-GIT stayed under it. Figure 8 depicts the concordance between QFT-GIT and QFT-Plus Tube TB2. On five occasions the QFT-GIT had values over the cutoff while at the same time TB2 stayed under it. Three times TB2 was over the cutoff while QFT-GIT was under it.

The ICC for the QFT-Plus, representing the proportion of the variance that is attributed to the variation between individuals, was 0.88. This correspondingly indicates that variance in the individuals was low.

### Active TB infection and chest X-ray results

None of the chest X-rays of participants with a positive IGRA-result showed signs of ongoing active TB. Despite the study physicians advice two of the participants refused to take their X-ray. As stated above, none of the participants showed clinical signs of ongoing active TB infection so it was assumed that no participant with active TB was taking part in the study.

The one participant formerly treated for active TB, but now being smear- and culturally negative, showed consistently positive results in both IGRAs.

## Discussion

Our study confirms the occurrence of conversions and reversions in both the well studied QFT-GIT and the new generation IGRA QFT-Plus. The QFT-GIT had a reversion rate of 3.2% and a conversion rate of 2.2%. Both rates were slightly higher for the QFT-Plus: 6.9% reversions and 4.3% conversions. Due to the small number of blood samples evaluated, the confidence intervals for the variability rates of the two IGRAs compared are large and overlap each other.

The overall agreement between the two IGRAs of 95.1% (155/163) was good (κ = 0.89). Admittedly, one must take into account that a regression-to-the-mean phenomenon might have led to the increased concordance during the successive visits. This is caused by random fluctuation around a true mean. Each time the same subject undergoes repeated measurement, extreme observations might be followed by ones that are closer to the subject’s true mean. This could lead to natural variation looking like real change in serial testing of the same subject [29]. Ringshausen et al. and Tagmouti et al. identified this phenomenon as an additional source of test variability in serial IGRA testing [8, 17], especially when IGRA positive subjects are longitudinal followed.

As all variations took place within a narrow range close to the manufacturers’ cutoff, the use of a borderline zone like already proposed for the QFT-GIT might as well be useful for the interpretation of changes around the cutoff in the QFT-Plus. For the QFT-GIT Ringshausen et al. in their review advocate the use of a borderline zone from 0.2–0.7 IU/ml. The authors suggest that subjects with borderline zone results and suspected infection should be retested before recommending preventive chemotherapy [9]. Using this definition for the QFT-Plus and defining a conversion as a cross passing of the borderline zone from below to above and a reversion as a cross passing from above the borderline zone to below the borderline zone, we did not observe a conversion or reversion. Applying this definition for conversions will reduce the number of preventive treatments needed when a positive QFT is observed in serial testing.

Test results of QFT-Plus for individuals were very similar, with a high ICC of 0.88.

Furthermore it is worth noticing that in four cases only the IFN-γ value of the antigen TB2 tube crossed the manufacturer’s cutoff leading to conversion and reversion in those participants. The overall number of inconsistent results is too low to suggest that tube TB2 was the sole cause of the higher rate of variability. This observation seems plausible because of a further source of variation but deserves further investigation in follow-up studies.

Overall, inconsistent IGRA results of less than 7% occurred, which is less frequently than what can be found in the literature. Ringshausen et al. described reversion rates of 22.1% to 71.4% and conversion rates of 0.7% to 14.4% in their systematic review [9].

In another systematic review Zwerling et al. found 7% to 70% of reversions and 11.6% to 21% of conversions in serial testing of healthcare workers (HCW) in high-incidence countries, and 11% to 33% of reversions and 1.8% to 14.4% of conversions in HCW serial testing in low-incidence countries [6].

As our numbers, especially occurrence of reversions, are lower, it is important to note that these studies were undertaken with data from screening of HCWs and that the interval between subsequent visits varied between the studies but were in most cases longer than our seven-day interval.

In a study undertaken in a similar timeframe to ours, but also using HCWs as its study population and five time points, Ringshausen et al. found 28.6% of inconsistent results (inconsistency in 10 out of 35 subjects). Comparing results of subsequent visits to baseline results, they had 50% of reversions (6 out of 12 initial positive subjects) and 17.4% of conversions (4 out of 23 initial negative subjects) [17].

King et al. in their variability study on a large cohort of U.S. HCWs investigating the other commercially available IGRA, the T-SPOT.TB, showed lower conversion rates: 0.8% conversions and 17.6% reversions (or 1.6% conversions and 26.4% reversions if including borderline zone results). Their study shows a strong correlation between the risk of conversion and the reported TB incidence rates of the state in which the HCWs were located [30]. The lower conversion rates in the study by King et al. could as well be inherent to the different assay that was used. Compared to the ELISPOT based assay the ELISA based assays like QFT-GIT and QFT-Plus might show higher variability due to the limited control of the number of T-cells in the vials. This was suggested by an assay comparison concerning Cytomegalovirus (CMV) infection comparing CMV ELISPOT and CMV QuantiFERON: Saldan et al. assume interassay differences like the comparison of CMI responses in a volume of blood in QuantiFERON assays versus a given number of cells in ELISPOT assays to be a potential source of variability [31].

Systematic testing of HCWs for LTBI is a main area of use for serial IGRA testing. As stated above, most studies on IGRA variability are undertaken on HCWs with longer intervals between blood collections. New TB exposure at intervals between the study visits might cause IGRA conversion. Zwerling et al. note in their systematic review that occupational TB risk factors correlate with IGRA positivity but that it remains unclear whether occupational TB exposure is associated with IGRA conversion [6]. In the following, Zwerling et al. ran different studies to evaluate the impact of occupational TB exposure on IGRA conversion. Such an association could not be shown in Canadian HCWs [24] but was present in another study with nursing students in India [32]. In order to eliminate TB exposure as a factor of IGRA variability during our study, intervals between blood collections were short and a cohort with low probability of recent TB contact was chosen. On the other hand untargeted systematically testing of low risk groups could lead to higher false positive rates [2]. So we chose a group of participants with a higher risk than the native population in Germany (German TB incidence 6.2:100,000): students with a migration background from countries with a TB incidence of at least 10 incident cases per 100,000 per year. As a matter of fact, all participants with an initial positive IGRA come from a country with high TB incidence ≧ 125:100,000 or from a country classified as a high TB burden country by the WHO. Therefore it is likely that the positive results at baseline were ‘true positive’ results. This might explain the low reversion rate we observed compared to the HCW studies mentioned above. However, no gold standard for LTBI testing exists to which IGRA results can be compared. As no active TB was found in our study, it is impossible to say if positives are ‘true positives’ in respect of LTBI.

Yet all 11 participants with a positive baseline result in one of the two IGRAs stayed positive in at least one of the two IGRAs through all four visits. One of those 11 students was treated for active TB and had consistently positive results in both IGRAs. It was shown in other studies that IGRA positivity can persist in people formerly infected with active TB after completion of antituberculosis chemotherapy and that IGRAs therefore are not a sufficient tool for monitoring antituberculosis chemotherapy [33]. It seems plausible that the other 10 participants with positive results in both IGRAs on the final visit were indeed true positives with regard to LTBI. All conversions in those participants were stable conversions.

Even the 12th participant that only had one positive QFT-Plus result on visit 3 and later reverted was born in a high-incidence country and reported a close contact to active TB over ten years ago (contact with a relative). The positive QFT-Plus result might indeed be an immunological footprint of that former contact but because of the unstable conversion and all-negative results in the other visits this case was not classified as LTBI.

Still it is important to notice that reversion does not mean that the previous QFT was false positive as suggested by a recent study from South Africa. Andrews et al. demonstrated that the predictive value of a positive IGRA result is higher than the predictive value of a negative test result, even if the test reverts to negative at the following control. Incident tuberculosis was 8-fold higher among QFT reverters than in participants with all negative QFT results [34].

### Strengths and limitations

Our study is the first study we are aware of that investigated variability of the new generation QFT-Plus in serial testing in a direct head-to-head comparison with QFT-GIT.

The number of participants tested was rather small (*n* = 41) but a total of 163 serial measurements were analyzed for each IGRA. These numbers align with similar studies on IGRA variability [17].

Choosing students with a migration background studying at a German university as our study subjects gave us an opportunity to evaluate the IGRAs in a cohort with higher LTBI risk than the native population in a low TB incidence setting. Therefore it is highly unlikely that our participants had new contact to active TB during the study period.

The students proved to be a reliable cohort with all of the participants attending their appointments punctually. The only missing result was caused by a blood sample that could not be analyzed. None of the participants dropped out of the study.

Letting individuals with a recent TST application (less than 3 or even 6 months prior first study visit) participate although other studies tend to exclude these individuals due to possible IGRA boosting [17, 24] might be a limitation of the study as well as a strength because we investigated all of the subjects without any censoring. Other sources also indicate no IGRA boosting following TST application. These studies advise against attributing IGRA positivity to possible boosting induced by a previous TST [8, 35].

## Conclusions

Although an initial recent study certifies the new generation IGRA QFT-Plus as delivering results with good sensitivity and specificity [22], our study could not confirm improvements in the area of test variability. Inclusion of an additional test tube elucidating CD8^+^ T-cell response can give rise to further variability. However, following our data the increase in variability was rather small due to the high agreement between QFT-GIT and QFT-Plus. Assuming that the additional tube used in the QFT-Plus will improve sensitivity, the similarity in variability suggests that QFT-Plus has the potential to be advantageous compared to its predecessor.

## Additional file

Additional file 1: Overview with exact IFN-γ values for all subjects at each visit and overall consistency. (PDF 67 kb)

## Abbreviations

BCG: Bacillus calmette-guérin
BGW: Institute for Statutory Accident Insurance and Prevention in the Health and Welfare Services
CI: Confidence interval
CMI: Cell-mediated immune response
CMV: Cytomegalovirus
CVcare: Center of Excellence for Health Services Research in Nursing
ECDC: European Centre for Disease Prevention and Control
ELISA: Enzyme-linked immunosorbent assay
FDA: U.S. Food and Drug Administration
GPR: Principles of Prevention and Rehabilitation Department
HCW: Healthcare worker
ICC: Intraclass correlation coefficient
IFN-γ: Interferon-gamma
IGRA: Interferon-gamma release assay
LTBI: Latent tuberculosis infection
NTM: Nontuberculous mycobacteria
QFT-GIT: QuantiFERON-TB Gold In-Tube
QFT-Plus: QuantiFERON-TB Plus
SD: Standard deviation
TB: Tuberculosis
TST: Tuberculin skin test
UKE: University Medical Center Hamburg-Eppendorf
WHO: World Health Organization
