# Supplementary material for: QFT-Plus: a plus in variability? – Evaluation of new generation IGRA in serial testing of students with a migration background in Germany
Source: J Occup Med Toxicol. 2017 Jan 5;12:1. doi: 10.1186/s12995-016-0148-z (PMC5216544; doi:10.1186/s12995-016-0148-z)
Supplement: Additional file 1: — Overview with exact IFN-γ values for all subjects at each visit and overall consistency. (PDF 67 kb) [file 12995_2016_148_MOESM1_ESM.pdf]

Additional table 1 Overview with exact IFN-ɣ values for all subjects at each visit and overall consistency.

Subject info			Visit 1		Visit 2		Visit 3		Visit 4		Overall trend	
No.	Sex	Age	QFT-GIT	QFT-Plus TB1/TB2	QFT-GIT	QFT-Plus TB1/TB2	QFT-GIT	QFT-Plus TB1/TB2	QFT-GIT	QFT-Plus TB1/TB2	Consistency QFT-GIT	Consistency QFT-Plus
1	F	25	pos. (>10)	pos. (>10/>10)	pos. (>10)	pos. (>10/>10)	pos. (>10)	pos. (>10/>10)	pos. (>10)	pos. (>10/>10)	Positive	Positive
2	M	25	pos. (7.22)	pos. (4.00/5.07)	pos. (7.01)	pos. (5.80/6.01)	pos. (7.34)	pos. (6.14/6.46)	pos. (5.85)	pos. (8.01/7.19)	Positive	Positive
3	M	28	pos. (6.20)	pos. (2.55/2.82)	pos. (3.83)	pos. (2.67/2.17)	pos. (5.73)	pos. (1.65/2.93)	pos. (4.24)	pos. (1.49/2.45)	Positive	Positive
4	M	34	pos. (4.41)	pos. (4.24/5.19)	pos. (7.34)	pos. (6.51/6.64)	pos. (3.85)	pos. (4.66/5.63)	pos. (2.77)	pos. (3.21/3.81)	Positive	Positive
5	M	26	pos. (1.38)	pos. (0.75/1.49)	pos. (0.92)	pos. (1.08/1.71)	pos. (1.41)	pos. (1.29/2.06)	pos. (2.20)	pos. (1.85/2.46)	Positive	Positive
6	F	26	pos. (1.30)	pos. (2.47/2.17)	pos. (5.53)	pos. (1.50/1.70)	pos. (6.26)	pos. (1.73/3.10)	pos. (3.91)	pos. (3.73/4.05)	Positive	Positive
7	F	26	pos. (0.81)	pos. (0.85/0.82)	pos. (1.44)	pos. (0.66/0.41)	pos. (1.41)	pos. (0.45/0.61)	pos. (1.05)	pos. (0.65/0.87)	Positive	Positive
8	M	36	pos. (0.71)	pos. (0.75/0.77)	pos. (1.05)	pos. (1.25/1,23)	pos. (0.88)	pos. (0.99/1.18)	pos. (0.62)	pos. (1.08/0.70)	Positive	Positive
9	M	32	pos. (0.40)	neg. (0.15/0.19)	pos. (0.62)	neg. (0.17/0.14)	pos. (0.66)	neg. (0.14/0.09)	pos. (0.65)	pos. (0.34/0.40)	Positive	Inconsistent
10	M	20	pos. (0.35)	neg. (0.24/0.20)	neg. (0.31)	pos. (0.50/0.47)	pos. (0.46)	pos. (0.55/0.73)	pos. (0.53)	pos. (0.79/0.69)	Inconsistent	Inconsistent
11	M	23	neg. (0.18)	pos. (0.32/0.64)	pos. (0.52)	neg. (0.11/0.12)	pos. (0.47)	pos. (0.46/0.40)	pos. (0.63)	pos. (0.56/0.61)	Inconsistent	Inconsistent
12	M	25	neg. (0.04)	neg. (0.08/0.08)	neg. (0.09)	neg. (0.05/0.14)	neg. (0.09)	pos. (0.16/0.36)	neg. (0.14)	neg. (0.21/0.24)	Negative	Inconsistent
13	F	23	neg. (0.02)	neg. (0.09/0.00)	neg. (0.26)	neg. (0.11/0.05)	neg. (0.18)	neg. (0.08/0.05)	neg. (0.18)	neg. (0.05/0.04)	Negative	Negative
14	M	25	neg. (0.11)	neg. (0.05/0.11)	neg. (0.06)	neg. (0.07/0.07)	missing	missing	neg. (0.06)	neg. (0.12/0.06)	Negative	Negative
15	M	23	neg. (0.02)	neg. (0.00/0.00)	neg. (0.03)	neg. (0.00/0.00)	neg. (0.09)	neg. (0.00/0.00)	neg. (0.00)	neg. (0.00/0.00)	Negative	Negative
16	F	26	neg. (0.07)	neg. (0.04/0.03)	neg. (0.02)	neg. (0.00/0.00)	neg. (0.00)	neg. (0.00/0.00)	neg. (0.03)	neg. (0.03/0.04)	Negative	Negative
17	F	24	neg. (0.07)	neg. (0.00/0.00)	neg. (0.02)	neg. (0.00/0.00)	neg. (0.00)	neg. (0.00/0.00)	neg. (0.03)	neg. (0.00/0.00)	Negative	Negative
18	F	25	neg. (0.04)	neg. (0.07/0.00)	neg. (0.00)	neg. (0.00/0.00)	neg. (0.00)	neg. (0.00/0.00)	neg. (0.01)	neg. (0.06/0.01)	Negative	Negative
19	M	26	neg. (0.02)	neg. (0.00/0.00)	neg. (0.00)	neg. (0.00/0.00)	neg. (0.03)	neg. (0.01/0.00)	neg. (0.06)	neg. (0.00/0.00)	Negative	Negative
20	M	25	neg. (0.00)	neg. (0.00/0.00)	neg. (0.02)	neg. (0.03/0.00)	neg. (0.00)	neg. (0.00/0.00)	neg. (0.03)	neg. (0.06/0.01)	Negative	Negative
21	F	30	neg. (0.05)	neg. (0.00/0.01)	neg. (0.03)	neg. (0.00/0.00)	neg. (0.03)	neg. (0.00/0.01)	neg. (0.00)	neg. (0.00/0.00)	Negative	Negative
22	M	27	neg. (0.00)	neg. (0.00/0.00)	neg. (0.03)	neg. (0.00/0.01)	neg. (0.00)	neg. (0.00/0.05)	neg. (0.00)	neg. (0.00/0.00)	Negative	Negative
23	F	21	neg. (0.00)	neg. (0.04/0.01)	neg. (0.00)	neg. (0.02/0.01)	neg. (0.01)	neg. (0.01/0.01)	neg. (0.01)	neg. (0.01/0.03)	Negative	Negative
24	M	25	neg. (0.00)	neg. (0.01/0.01)	neg. (0.00)	neg. (0.02/0.00)	neg. (0.00)	neg. (0.04/0.04)	neg. (0.01)	neg. (0.00/0.00)	Negative	Negative
25	F	22	neg. (0.00)	neg. (0.00/0.04)	neg. (0.00)	neg. (0.01/0.00)	neg. (0.00)	neg. (0.00/0.00)	neg. (0.00)	neg. (0.00/0.00)	Negative	Negative
26	F	31	neg. (0.01)	neg. (0.02/0.02)	neg. (0.01)	neg. (0.00/0.04)	neg. (0.00)	neg. (0.00/0.01)	neg. (0.00)	neg. (0.01/0.03)	Negative	Negative
27	F	24	neg. (0.02)	neg. (0.00/0.01)	neg. (0.01)	neg. (0.01/0.01)	neg. (0.03)	neg. (0.01/0.01)	neg. (0.01)	neg. (0.00/0.01)	Negative	Negative
28	F	22	neg. (0.00)	neg. (0.00/0.00)	neg. (0.01)	neg. (0.00/0.01)	neg. (0.00)	neg. (0.00/0.01)	neg. (0.00)	neg. (0.01/0.03)	Negative	Negative
29	F	25	neg. (0.00)	neg. (0.02/0.02)	neg. (0.00)	neg. (0.00/0.02)	neg. (0.01)	neg. (0.02/0.02)	neg. (0.00)	neg. (0.01/0.00)	Negative	Negative
30	F	24	neg. (0.02)	neg. (0.00/0.01)	neg. (0.01)	neg. (0.01/0.01)	neg. (0.00)	neg. (0.01/0.01)	neg. (0.01)	neg. (0.00/0.00)	Negative	Negative
31	M	23	neg. (0.00)	neg. (0.00/0.00)	neg. (0.01)	neg. (0.01/0.01)	neg. (0.02)	neg. (0.00/0.00)	neg. (0.02)	neg. (0.00/0.00)	Negative	Negative
32	F	24	neg. (0.00)	neg. (0.00/0.00)	neg. (0.00)	neg. (0.00/0.00)	neg. (0.00)	neg. (0.00/0.02)	neg. (0.00)	neg. (0.01/0.01)	Negative	Negative
33	F	24	neg. (0.00)	neg. (0.00/0.00)	neg. (0.00)	neg. (0.00/0.00)	neg. (0.00)	neg. (0.00/0.00)	neg. (0.01)	neg. (0.01/0.02)	Negative	Negative
34	M	27	neg. (0.00)	neg. (0.01/0.00)	neg. (0.01)	neg. (0.00/0.00)	neg. (0.00)	neg. (0.01/0.00)	neg. (0.00)	neg. (0.00/0.00)	Negative	Negative
35	M	24	neg. (0.00)	neg. (0.00/0.00)	neg. (0.01)	neg. (0.01/0.01)	neg. (0.01)	neg. (0.01/0.01)	neg. (0.01)	neg. (0.00/0.00)	Negative	Negative
36	F	27	neg. (0.00)	neg. (0.01/0.00)	neg. (0.01)	neg. (0.00/0.01)	neg. (0.01)	neg. (0.00/0.00)	neg. (0.01)	neg. (0.01/0.00)	Negative	Negative
37	F	23	neg. (0.00)	neg. (0.01/0.00)	neg. (0.01)	neg. (0.01/0.01)	neg. (0.00)	neg. (0.00/0.00)	neg. (0.00)	neg. (0.00/0.00)	Negative	Negative
38	F	33	neg. (0.00)	neg. (0.01/0.01)	neg. (0.00)	neg. (0.00/0.00)	neg. (0.00)	neg. (0.01/0.01)	neg. (0.00)	neg. (0.00/0.00)	Negative	Negative
39	F	22	neg. (0.00)	neg. (0.00/0.00)	neg. (0.01)	neg. (0.00/0.00)	neg. (0.01)	neg. (0.00/0.00)	neg. (0.00)	neg. (0.00/0.00)	Negative	Negative
40	M	20	neg. (0.00)	neg. (0.01/0.00)	neg. (0.00)	neg. (0.00/0.00)	neg. (0.00)	neg. (0.00/0.00)	neg. (0.00)	neg. (0.00/0.00)	Negative	Negative
41	F	25	neg. (0.00)	neg. (0.00/0.00)	neg. (0.01)	neg. (0.00/0.00)	neg. (0.00)	neg. (0.00/0.00)	neg. (0.00)	neg. (0.00/0.00)	Negative	Negative
